# Animal Toxins: Biodiscovery, Mechanistic Insights and Translational Potential

**DOI:** 10.3390/toxins16030130

**Published:** 2024-03-01

**Authors:** Tim Lüddecke, Simon Blank

**Affiliations:** 1 Department of Bioresources, Fraunhofer Institute for Molecular Biology and Applied Ecology, Ohlebergsweg 12, 35392 Giessen, Germany; 2 LOEWE-Centre for Translational Biodiversity Genomics, Senckenberganlage 25, 60325 Frankfurt am Main, Germany; 3 Center of Allergy & Environment (ZAUM), Technical University of Munich, School of Medicine & Helmholtz Munich, German Research Center for Environmental Health, 85764 Munich, Germany

Nature abounds with an unprecedented diversity of biomolecular innovation. Among those, venoms rank among the most valuable yet simultaneously devastating mechanisms. As key evolutionary adaptions, venoms and poisons evolved more than 100 times convergently across the animal kingdom and serve the three primary functions of hunting, defense and intraspecific competition, plus a series of secondary functions [1,2,3]. Their bioactive components are referred to as toxins and belong to the most potent and selective natural products known [4]. While this potency and selectivity, on the one hand, can cause potentially life-threatening envenoming (e.g., in the context of the global snakebite crisis [5,6,7]), the toxins of animal venoms and poisons may also be repurposed into biomedical, biotechnological or agricultural products [8,9,10]. Accordingly, animal venoms and poisons have been targeted in several biodiscovery programs, yet the majority of taxa and their biomolecular arsenal remain virtually unstudied [11]. This stems from persistent methodological limitations that were only recently overthrown by the emergence of modern venomics approaches that utilize cutting-edge systems biology, bioinformatics and biotechnology to unveil venom/poison composition and function [12].

Thanks to the modern venomics revolution, toxin cocktails from essentially all extant animals can be identified, starting from minuscule amounts of sample material [12]. Consequently, venom and poison compositions from several hitherto neglected aquatic, marine, and terrestrial animals have been disentangled, and their toxins could be functionally characterized [13,14,15,16,17]. At the same time, these more profound insights into previously under- or unstudied animal lineages broadened the array of potential applications, particularly in the context of biomedical uses. A wide range of novel toxin families, chemical modifications, and molecular interplays have also been unveiled [2,18,19,20]. Moreover, in addition to the traditional cardiovascular and neurological applications of animal toxins, a growing body of studies pinpoints potential, e.g., in the battle against multi-drug resistant bacteria, viruses, and parasites [21,22,23,24,25]. Overall, it must be concluded that in terms of taxonomic coverage, functional understanding and translational potential of toxins from across the animal kingdom, only little is known thus far.

This Special Issue intends to shed light on many of the understudied compositional, functional and applied aspects of animal toxins in venom and poison cocktails. For this purpose, research and review articles targeting a wide range of questions related to the above conundrum and focusing on very distinct organisms have been welcomed. In total, this collection comprises eight articles.

The first contribution of our Special Issue is from Alzeer and colleagues and investigates the activity of the ant venom peptide Pilosulin-3. The authors specifically investigated whether or not the peptide may serve in breast cancer therapy in synergism with radiation.

The second contribution to this Special Issue focuses on venom peptides from the tarantula *Lasiodora klugi*. In their work, Ahmed and colleagues applied bioassay-guided screening of arachnid venoms to identify *L. klugi* peptides as a potential weapon to battle invasive *Aedes aegypti* mosquitoes.

Parasitoid wasps belong to the least studied venomous animals on earth, and the third contribution, authored by Yu and colleagues, provides novel insights on this matter. Specifically, the authors developed a novel artificial host-based venom collection method and used it to unveil the venom composition of the parasitoid *Habrobracon hebetor*.

The fourth contribution to our Special Issue is authored by Lüddecke and colleagues and investigates the bioactivity of an entire family of linear cytolytic peptides from wolf spiders, genus *Lycosa*. Via in vivo and in vitro activity assays with synthesized components, the authors show that these peptides have antimicrobial activity and may serve to protect the venom gland against infection.

Contribution five stems from Fischer and colleagues and examines multifunctional venom compounds from the assassin bug *Psytalla horrida*. In their exciting study, the authors demonstrate, via chromatography-based fractionation and bioassays, that *P. horrida* venom components affect cell viability, bacterial growth and, among others, insect neuronal calcium homeostasis. Their study adds to the growing body of evidence that assassin bug venoms are functionally of great complexity.

Snakebite is a neglected tropical disease, and detailed mechanistic studies are needed to support emergency care. In their herein-presented work, contribution six to our Special Issue, the authors around Figueiredo carried out an experimental study in murine models to investigate the effects of *Crotalus durissus cascavella* on venom-induced pulmonary impairment. Their work underpins the importance of prompt snakebite treatment to protect the pulmonary system from venom-induced damage.

In contribution seven, authored by Hurka and colleagues, novel insights into the potential of ant venom peptides to battle infectious diseases are presented. Via in vitro bioactivity screenings on transcriptome-mined peptides from myrmicine ant venom, the authors show that several peptides are active against pathogenic bacteria yet relatively non-toxic towards human cells. Their work paves the way for future investigations looking into ant venom-derived antibiotics.

The last contribution to our Special Issue is a review paper authored by Nagy and colleagues. In their comprehensive overview, the authors present a birds-eye perspective on zootoxins and their importance for domestic animals.
